# Prediction of hand, foot, and mouth disease epidemics in Japan using a long short-term memory approach

**DOI:** 10.1371/journal.pone.0271820

**Published:** 2022-07-28

**Authors:** Kazuhiro Yoshida, Tsuguto Fujimoto, Masamichi Muramatsu, Hiroyuki Shimizu

**Affiliations:** 1 Department of Virology II, National Institute of Infectious Diseases, Tokyo, Japan; 2 Department of Fungal Infection, National Institute of Infectious Diseases, Tokyo, Japan; Hanyang University, REPUBLIC OF KOREA

## Abstract

Hand, foot, and mouth disease (HFMD) is a common febrile illness caused by enteroviruses in the *Picornaviridae* family. The major symptoms of HFMD are fever and a vesicular rash on the hand, foot, or oral mucosa. Acute meningitis and encephalitis are observed in rare cases. HFMD epidemics occur annually in Japan, usually in the summer season. Relatively large-scale outbreaks have occurred every two years since 2011. In this study, the epidemic patterns of HFMD in Japan are predicted four weeks in advance using a deep learning method. The time-series data were analyzed by a long short-term memory (LSTM) approach called a Recurrent Neural Network. The LSTM model was trained on the numbers of weekly HFMD cases in each prefecture. These data are reported in the Infectious Diseases Weekly Report, which compiles the national surveillance data from web sites at the National Institute of Infectious Diseases, Japan, under the Infectious Diseases Control Law. Consequently, our trained LSTM model distinguishes between relatively large-scale and small-scale epidemics. The trained model predicted the HFMD epidemics in 2018 and 2019, indicating that the LSTM approach can estimate the future epidemic patterns of HFMD in Japan.

## 1. Introduction

Enteroviruses belong to the *Picornaviridae* family and are categorized into 15 species (*Enterovirus A–L* and *Rhinovirus A–C*). More than 100 types of enteroviruses have been identified in humans. They are mainly transmitted by the fecal–oral route or respiratory infection route. A wide variety of clinical symptoms present in enterovirus infections, such as common cold-like illnesses, acute bronchitis, rashes, encephalitis, and paralysis [1–4].

Hand, foot, and mouth disease (HFMD) is a common febrile illness caused by enteroviruses. The main symptoms of HFMD are fever and a vesicular rash on the hand, foot, or oral mucosa, but acute meningitis and encephalitis can occur in rare cases [5, 6]. The most common HFMD pathogens are enterovirus A71 (EV-A71) and coxsackievirus A16 (CV-A16) [7]. In recent years, coxsackievirus A6 (CV-A6) has joined the conventional causative viruses (EV-A71 and CV-A16) as a major causative virus worldwide [8–16]. HFMD epidemics occur annually in Japan, usually in the summer season and usually in children. Large-scale epidemics, mainly associated with CV-A6 [17], have occurred every two years since 2011. In Japan, the prevalence of HFMD and herpangina is monitored at pediatric sentinel sites and reported in the Infectious Disease Weekly Report (IDWR) of the National Institute of Infectious Diseases.

The estimate of the dynamics of HFMD epidemics was conducted in previous studies using the computational algorithm [7, 18–28]. The incidence of HFMD was estimated by using the past epidemics in China [18–24], Thailand [25], Singapore [26], and Japan [7, 27, 28]. In Japan, the time-series susceptible-infected-recovered model [7], the latitude-based approach [27], or the maximum entropy method and the least squares fitting method [28] were used for validating the availability of the models. These studies predicted the dynamic of HFMD epidemic throughout the year in Japan by each model.

To predict the occurrence of HFMD in Japan, we utilize the characteristics of deep learning. Deep learning algorithms are based on neural networks [29], imitation models that mimic the networks of neurons and synapses in the brain. In a neural network, neurons are described as nodes and synapses are represented by the strengths of the connections between nodes. The “nodes” and “synapses” are up-dated during the learning process, building a thinking pattern for problem solving. Among the various types of neural networks are recurrent neural networks (RNNs), convolutional neural networks, feed-forward neural networks, and restricted Boltzmann machines [29–32]. RNNs are suitable for processing time-series data. An RNN has a return value by which a new calculation can refer to the previous calculation process. The representative RNN model is long short-term memory (LSTM), which returns a value called “cell” and calculates the output referring to the past calculation process in the cell [33, 34].

In this study, we trained an LSTM model on HFMD case numbers extracted from the pediatric sentinel sites reported in the IDWR, Japan. Using the trained model, we predicted the epidemic patterns of HFMD in 2018 and 2019 before the actual prevalence of HFMD was revealed, and demonstrated that when the number of reported HFMD cases increases in a certain prefecture, the likelihood of large-scale HFMD outbreaks throughout Japan also increases by showing how the LSTM model learned the IDWR’s data as the characteristic of machine learning. This study contributed the prediction of HFMD outbreaks in Japan therefore the application for public health measures to minimize the large HFMD outbreaks nationwide was expected.

## 2. Materials and methods

### 2.1. Statistical data

We analyzed the weekly sentinel data of pediatric HFMD (in the category of number of patients) per prefecture recorded in the IDWR. The IDWR was obtained from websites of the National Institute of Infectious Disease (https://www.niid.go.jp/niid/en/idwr-e.html; SENTINEL-REPORTING DISEASES (WEEKLY), which records the number of cases in the current week by prefecture in the Surveillance Data).

### 2.2. LSTM

**Fig 1:**
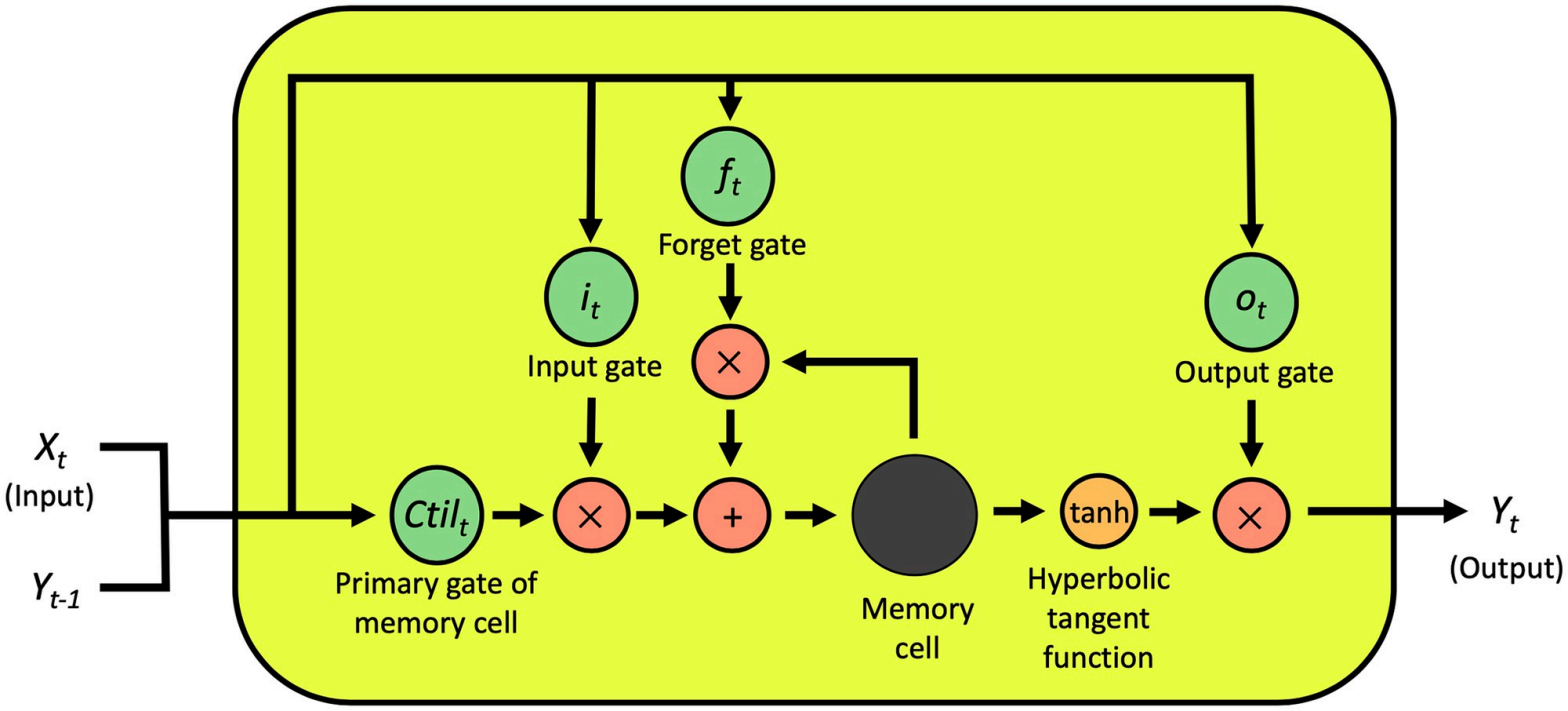
Summary of LSTM model. The formula of each step was described in 2.2. LSTM section.

The LSTM model was programed as previously described [33, 34] and is a kind of RNN, which is available for using time series data. The LSTM model contains “cell” which is the structure holding the characteristic of the past data as numeric value. The process, that the input is calculated by using the cell and the result is outputted, is basic structure of LSTM model [33]. Additionally, the circuit changing the numeric value in the cell for reflecting the characteristic of the new trend to the output is called as forget gate [34]. In this study, we used the model, which the forget gate was added into the basic structure of LSTM (Fig 1). The forget gate (*f*_*t*_), input gate (*i*_*t*_), primary gate of memory cell (*Ctil*_*t*_), memory cell (*C*_*t*_), output gate (*o*_*t*_), and output (*y*_*t*_) of the LSTM model were respectively computed as follows:

$$f_{t}=\delta \left(W_{f}\cdot \left[y_{t-1},\;x_{t}\right]+b_{f}\right),$$ (1)

$$i_{t}=\delta \left(W_{i}\cdot \left[y_{t-1},x_{t}\right]+b_{i}\right),$$ (2)

$$Ctil_{t}=\text{tanh}\left(W_{c}\cdot \left[y_{t-1},x_{t}\right]+b_{c}\right),$$ (3)

$$C_{t}=f_{t}\;\times \;C_{t-1}+i_{t}\;\times \;Ctil_{t},$$ (4)

$$o_{t}=\delta \left(W_{o}\cdot \left[y_{t-1},x_{t}\right]+b_{o}\right),$$ (5)

$$y_{t}=o_{t}\;\times \;\text{tanh}\left(C_{t}\right).$$ (6)

The symbol of *x*, *W*, and *b* were input, weight parameter, and threshold parameter, respectively. The input and the previous output were multiplied by weight parameter respectively, and threshold parameter was added in *f*_*t*_, *i*_*t*_, *Ctil*_*t*_, or *o*_*t*_ step. The signals in *f*_*t*_, *i*_*t*_, *Ctil*_*t*_, *o*_*t*_, or *y*_*t*_ step were converted to an activated signal by an activation function (a sigmoidal function; σ or hyperbolic tangent function; tanh). The input was normalized and standardized.

### 2.3. Confirming the appropriate input quantity by simulating the HFMD epidemic of 2015

The LSTM model was trained on pediatric sentinel weekly data by prefecture over the 1999–2014 period. For training, we inputted the epidemiological data of 1 to 6 consecutive weeks into the LSTM model. The model outputted the total number of patients in the following week and also compared the output value with the actual number of patients for training purposes. We then inputted the data of the five weeks of 2015 to the trained model, which simulated the total number of patients in 2015 using its learned approach. The input was again normalized and standardized. The maximum and minimum output values were adjusted to the maximum and minimum number of patients in 2015, respectively.

### 2.4. Construction of the trained LSTM model presenting the epidemic pattern of HFMD in 2014–2017

When constructing the trained model, the LSTM model received five weeks of data and outputted the total number of patients four weeks later. The output was simulated from the pediatric sentinel data collected over the 1999–2013 period. Based on the 2014–2017 data, we then simulated the total number of HFMD patients until the epidemic peaked in week 31. During this period, we judged that the simulation results described the actual epidemic. The input was normalized and standardized. The maximum output value in 2017 and the minimum output value in 2014–2017 were adjusted to the maximum number of patients in 2017 and 0, respectively. The output of the simulation results could then be adjusted such that the number of patients was between 0 and the maximum output in the most recent year.

### 2.5. Prediction of the epidemic pattern in 2018 and 2019

The three independently trained LSTM models were named Trials 10, 26, and 46. The trained models were provided with consecutive five weeks of data and outputted the total number of HFMD patients four weeks later (at week 25). The input was normalized and standardized. The maximum output value in 2019 and the minimum output value in 2014–2019 were adjusted to the maximum number of patients in 2019 and 0, respectively. The output value of the simulation results was then adjusted such that the number of patients was between 0 and the maximum output value in the most recent year.

### 2.6. Contribution of each prefecture’s epidemic against the nationwide HFMD outbreak of 2015

We replaced a number of HFMD patients per prefecture in the data of 2014 and 2015 aggregated in the IDWR reports, and inputted the modified data to the three independently trained models for simulating the total number of HFMD patients. The input data covered five weeks and the trained models outputted the total number of HFMD patients four weeks later. The input was normalized and standardized. We also summed the differences in the simulation results (weeks 9–31) obtained before and after modifying the input data. The actual numbers of HFMD patients by prefecture in 2015 were displayed as a heat map.

### 2.7. Root mean squared error (RMSE)

The accuracy of the simulation result was evaluated by the RMSE, defined as

$$RMSE=\sqrt{\frac{1}{n}\sum_{t=1}^{n}{\left(Y_{t}-y_{t}\right)}^{2}}.$$ (7)

In this expression, *Y*_*t*_ is the actual number of HFMD patients (standardized and normalized) and *y*_*t*_ denotes the simulation results. The RMSE defines the size of the prediction error in the output of the trained LSTM model.

### 2.8. Coefficient of determination (R^2^)

The accuracy of the simulation result was also evaluated by the R^2^, defined as

$$R^{2}=1-\frac{\sum_{t=1}^{n}{\left(y_{t}-\hat{y_{t}}\right)}^{2}}{\sum_{t=1}^{n}{\left(y_{t}-\bar{y}\right)}^{2}}$$ (8)

The *y*_*t*_ is the actual number of patients and $\bar{y}$ is the average of the *y*_*t*_. The $\hat{y_{t}}$ denotes the simulation results. The scale of the *y*_*t*_ and the $\hat{y_{t}}$ were matched for the calculation of R^2^. The R^2^ defines how the simulation results can be explained by the actual number of patients.

## 3. Results

### 3.1. Simulation of HFMD epidemic pattern in 2015 for confirming the appropriate input quantity

**Fig 2:**
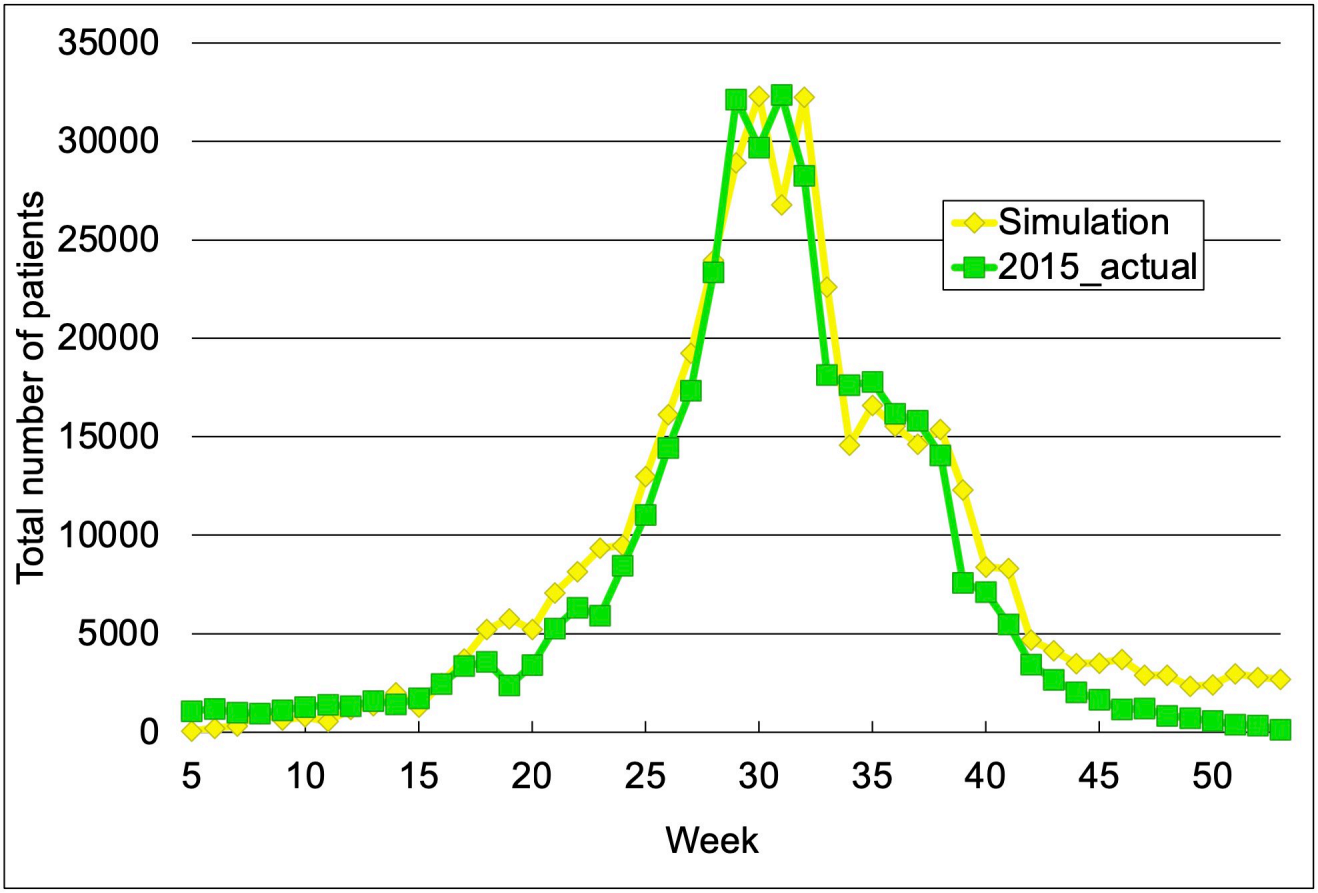
Simulation result of HFMD epidemic in 2015 (using 5 weeks of data as input). The maximum and minimum output values were adjusted to the maximum and minimum number of patients in 2015, respectively.

To confirm that the input quantity maximized the simulation performance, we simulated a number of HFMD patients in 2015 based on the IDWR reports of 1999–2014 (Fig 2). The LSTM model was trained on the weekly pediatric sentinel data by prefecture during the 1999–2014 period, extracted from the IDWR in Japan. More specifically, we inputted the number of HFMD patients over 1–6 consecutive weeks into the LSTM model. The model then outputted the total number of patients in the following week. The outputs of the LSTM model fairly matched the actual number of HFMD cases. To estimate the epidemic pattern in 2015, we inputted the data of 1–6 weeks in 2015 to the trained LSTM model. The model then simulated the total number of HFMD patients in 2015 using its learned method.

The simulation results almost matched the actual prevalence of HFMD cases. Among the 1–6 weeks of data input, five weeks of data achieved the best correspondence between the simulated and actual results (RMSE = 8.53 × 10^−7^, R^2^ = 0.944, and the evaluated period was week 5–53). Concretely, the simulated number of patients increased from around week 15, peaked in weeks 30–32, and decreased from week 33.

### 3.2. Simulation of HFMD epidemic 2–5 weeks after the data input period

**Fig 3:**
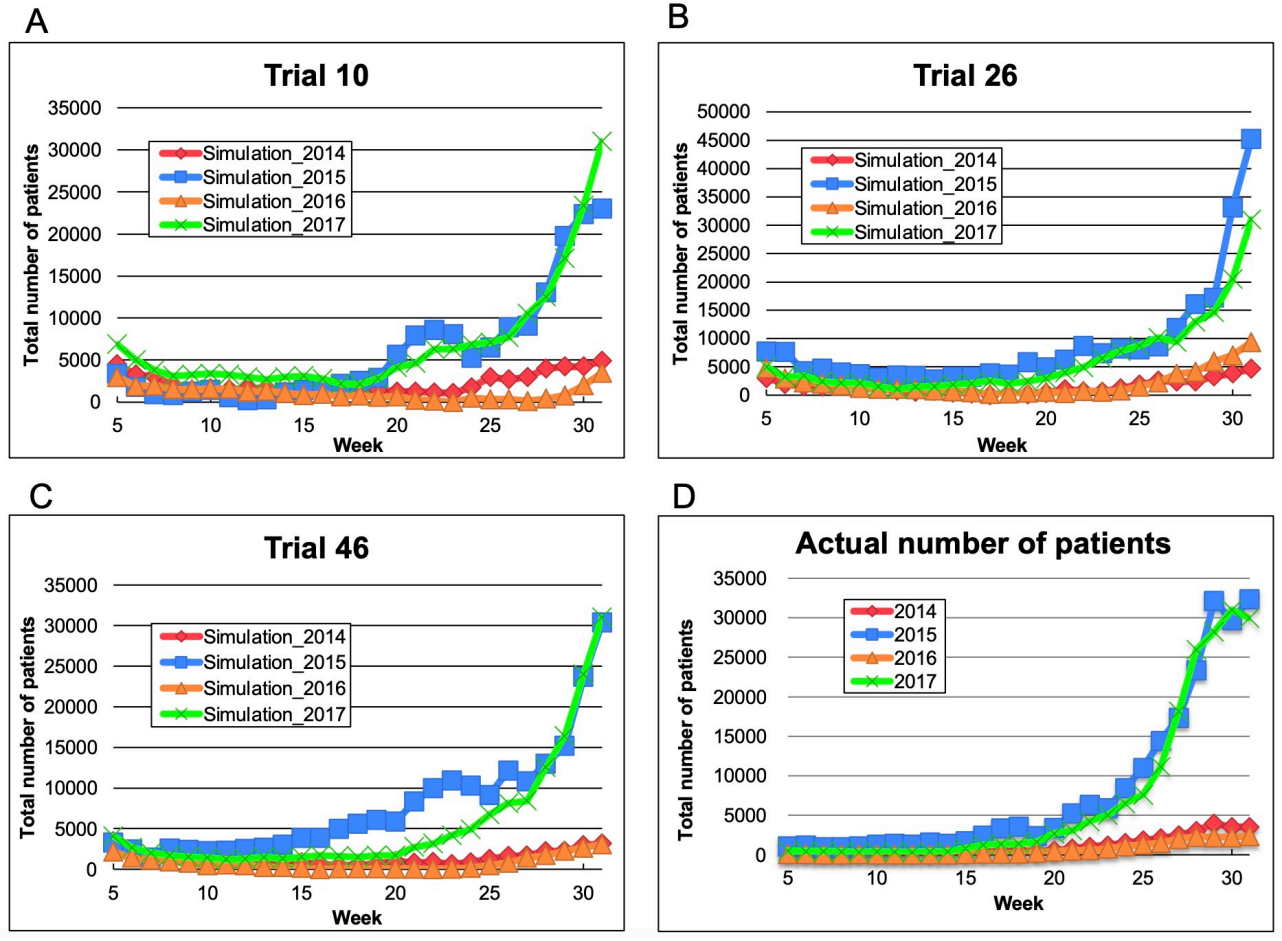
Epidemic patterns in 2014–2017 simulated by the three independently constructed models (obtained in the top-performing trials). The maximum output value in 2017 and the minimum output value in 2014–2017 were adjusted to the maximum number of patients in 2017 and 0, respectively: (A) trial 10; (B) trial 26; (C) trial 46; (D) actual number of HFMD patients. Diamonds: 2014; squares: 2015; triangles: 2016; crosses: 2017.

**Fig 4:**
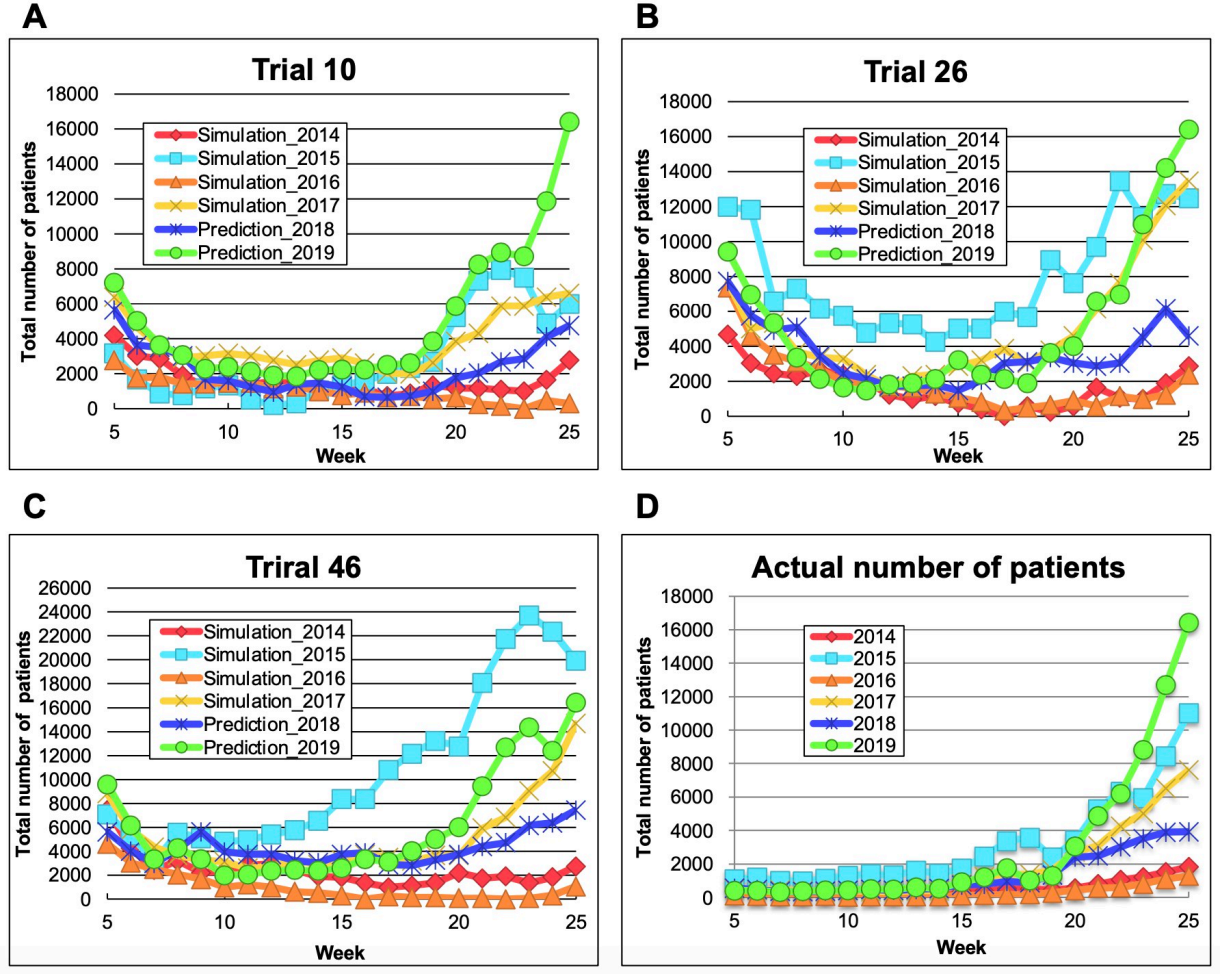
Epidemic patterns in 2018 and 2019 predicted by the three independently trained models (in trials 10, 26, and 46). (A–C): The maximum output value in 2019 and the minimum output value in 2014–2019 were adjusted to the maximum number of patients in 2019 and 0, respectively; (D) Actual numbers of patients until week 25.

To verify that the LSTM model could simulate the near-future increase in the number of HFMD patients, we simulated the number of HFMD cases at 2–5 weeks after the data input period in 2015 (see S1 Fig). The model was trained on five weeks of consecutive data over the 1999–2014 period as described for Fig 2 in the previous subsection. The model outputted the total number of patients 2–5 weeks later. In the 2015 simulation, the number of patients 2–5 weeks later was computed by the learned approach. The simulation results one week later (Fig 2) and 2–4 weeks later better matched the actual results than the simulation result of five weeks later (the RMSEs and the R^2^ are listed in S1 and S2 Tables). Therefore, the model could accurately simulate the number of HFMD patients up to four weeks after the data input period.

### 3.3. Construction and validation of the trained LSTM model presenting the epidemic patterns of 2014–2017

To predict the epidemic patterns of HFMD in 2018 and 2019, we trained the LSTM model to simulate the epidemic patterns of HFMD over the 2014–2017 period (Fig 3). During training, the model was inputted with five consecutive weeks of data over the 1999–2013 period and outputted the total number of patients four weeks later. To judge whether the model can describe the true epidemic, we simulated the total number of HFMD patients during 2014–2017 until the epidemic peaked in week 31. Tables 1 and 2 gave the RMSEs and the R^2^ of the total numbers of HFMD patients from week 1 to week 31 in the years 2014–2017 predicted in the best-performance trials of the three independently trained LSTM models (Trials 10, 26, and 46). The epidemic curves described by the trained models were similar to those of the actual epidemic. The simulation results of 2015 and 2017 showed sharp increases from around week 25, which were absent in the simulations of 2014 and 2016. Therefore, the epidemic trends were simulated one month before the epidemic peak.

**Table 1:** RMSEs of the simulation over the 2014–2017 period ^a^.

	2014	2015	2016	2017
**Trial 10**	5.62 × 10^−7^	5.38 × 10^−6^	7.36 × 10^−7^	2.46 × 10^−6^
**Trial 26**	6.00 × 10^−7^	6.53 × 10^−6^	5.22 × 10^−7^	6.97 × 10^−6^
**Trial 46**	9.11 × 10^−7^	7.42 × 10^−6^	4.56 × 10^−7^	7.16 × 10^−6^

^a^ Calculated using the summed differences in weeks 5–52 or weeks 5–53

**Table 2:** R^2^ of the simulation over the 2014–2017 period ^a^.

	2014	2015	2016	2017
**Trial 10**	-0.493	0.787	-1.389	0.785
**Trial 26**	0.408	0.741	-6.611	0.776
**Trial 46**	0.307	0.779	0.257	0.811

^a^ Calculated using the summed differences in weeks 5–31

### 3.4. Prediction of epidemic patterns of HFMD in 2018 and 2019

**Fig 5:**
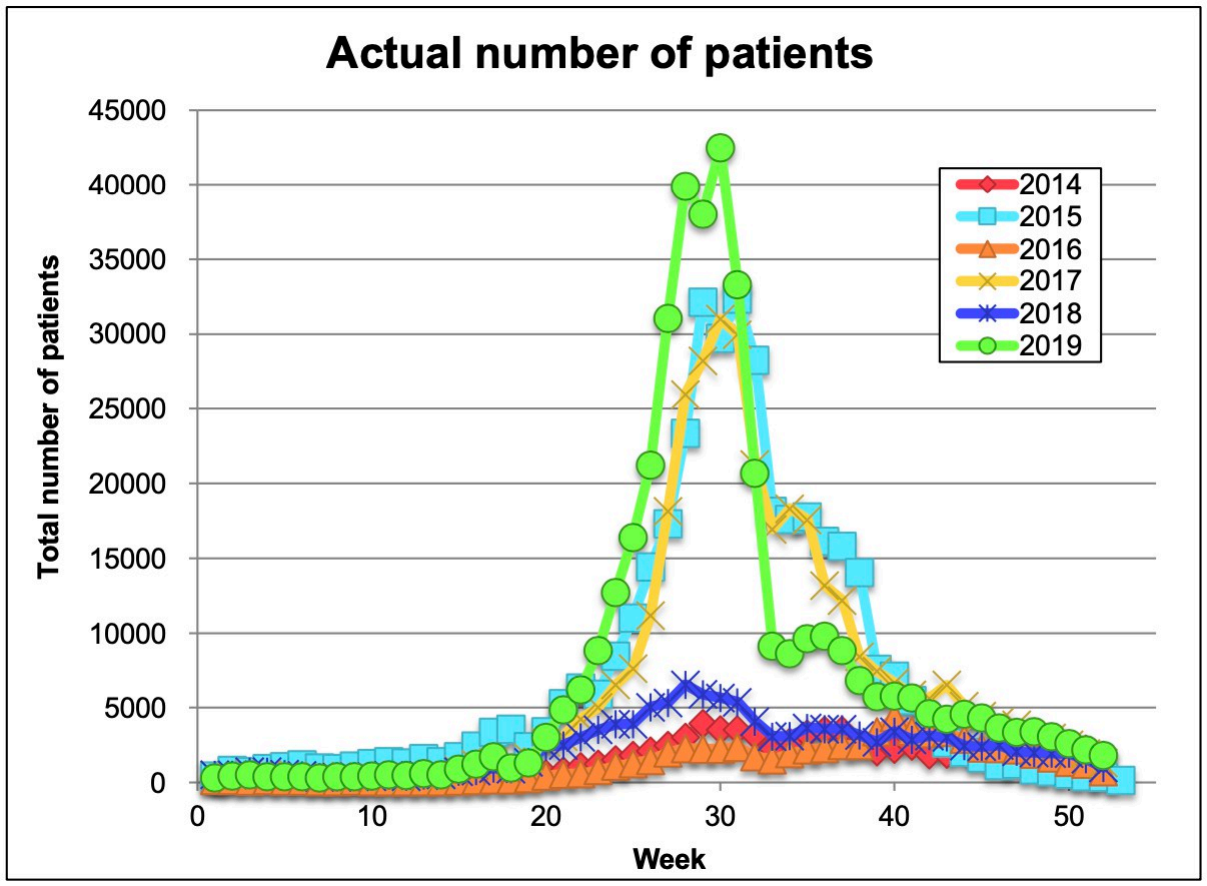
Actual epidemic patterns in 2014–2019. Actual numbers of patients in weeks 1–52 or 1–53.

To check whether the model can predict a yet-unreported HFMD epidemic, we predicted the epidemic patterns of HFMD in 2018 and 2019 using the three independently trained LSTM models (Fig 4). When predicting the 2018 and 2019 epidemics, we employed the method used for training the LSTM model. The three trained models predicted the epidemic pattern of 2018, which was slightly larger in scale than the 2014 and 2016 epidemics. The 2018 predictions were consistent with the actual HFMD patient numbers until week 25 (Fig 5). The three trained models also predicted the epidemic pattern of 2019, which was identical in scale to the 2015 and 2017 epidemics. The scales of the predicted and actual case numbers in 2019 were well matched. The RMSEs and the R^2^ of the 2018 and 2019 predictions are listed in Tables 3 and 4, respectively. Judging from these results, the trained LSTM model could predict the epidemic patterns of HFMD in 2018 and 2019.

**Table 3:** RMSEs of the 2018 and 2019 predictions ^a^.

	2018	2019
**Trial 10**	2.94 × 10^−7^	6.66 × 10^−7^
**Trial 26**	2.09 × 10^−7^	8.53 × 10^−7^
**Trial 46**	1.43 × 10^−7^	2.18 × 10^−6^

^a^ Calculated using the summed differences in weeks 6–25

**Table 4:** R^2^ of the 2018 and 2019 predictions ^a^.

	2018	2019
**Trial 10**	-0.109	0.758
**Trial 26**	-2.632	0.715
**Trial 46**	-4.454	0.456

^a^ Calculated using the summed differences in weeks 6–25

### 3.5. Influence size of each prefecture’s epidemic against the nationwide HFMD outbreak in Japan

**Fig 6:**
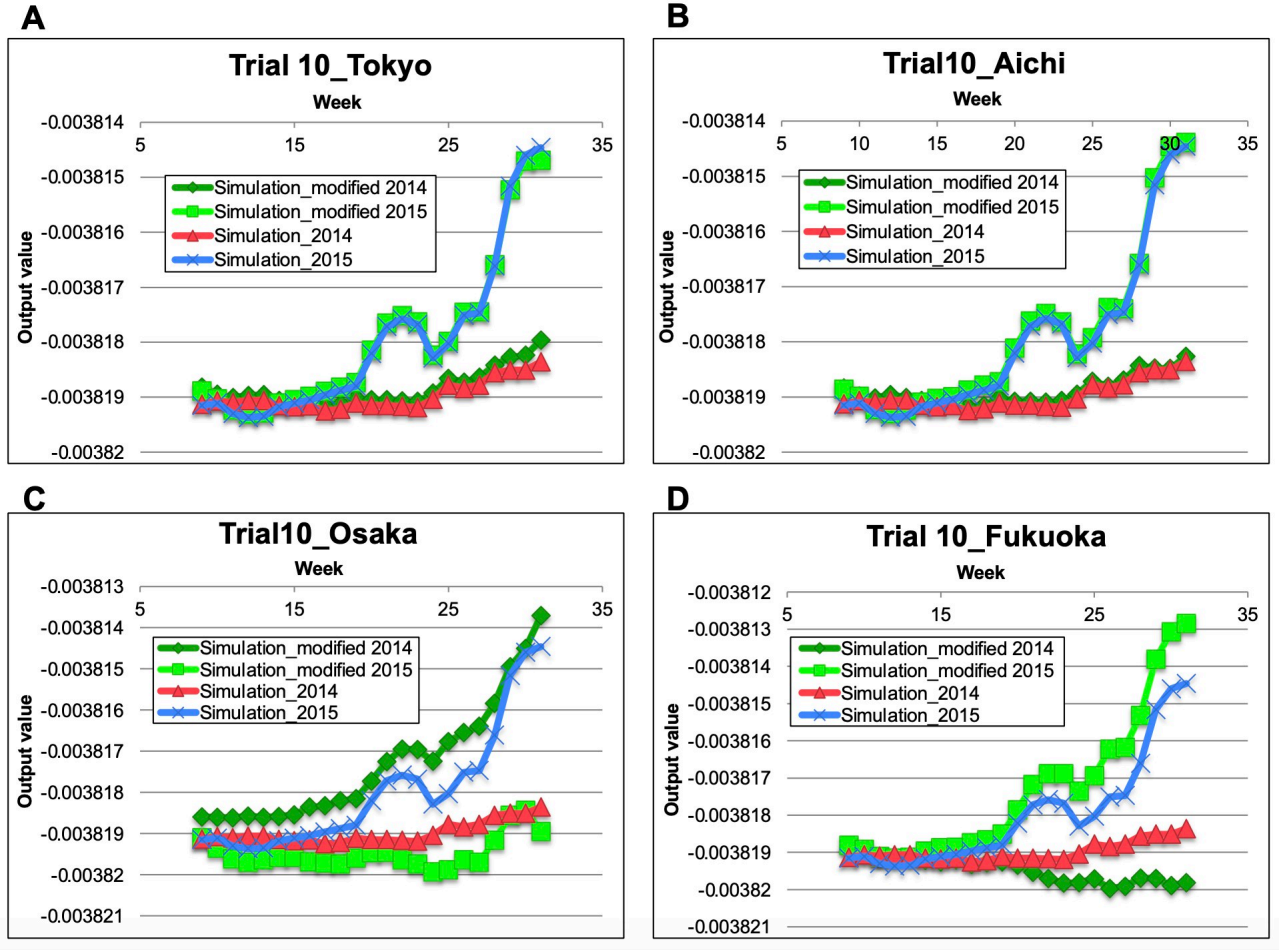
Example of simulation results of each prefecture’s epidemic by using the modified data. The three independently trained models were inputted with the pediatric sentinel data of 2014 and 2015 after replacing some data in Tokyo (A), Aichi (B), Osaka (C), and Fukuoka (D). Diamonds: modified 2014 data, squares: modified 2015 data, triangles: original 2014 data, crosses: original 2015 data.

**Fig 7:**
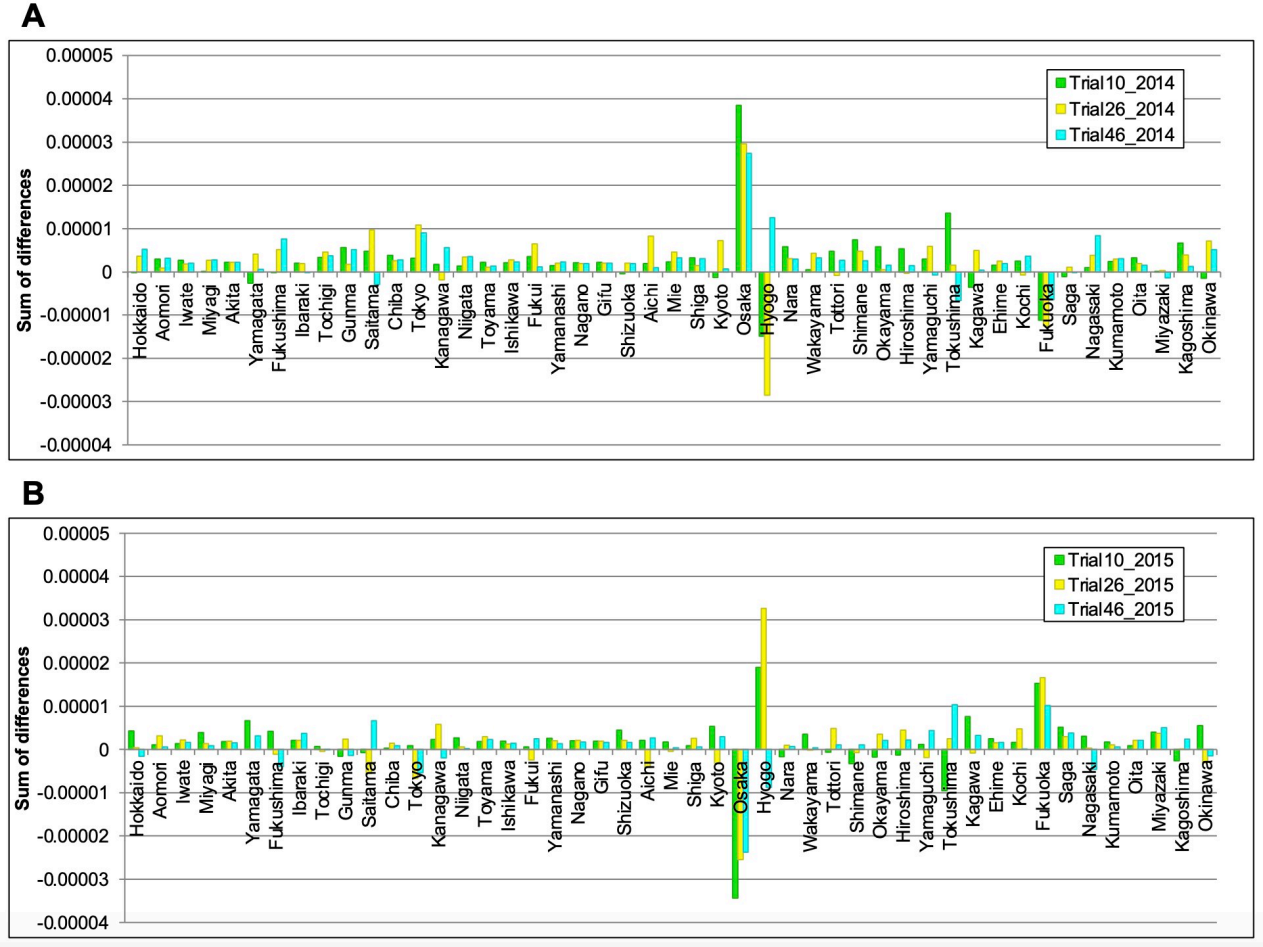
Size of influence of each prefecture’s epidemic against the HFMD outbreak of 2015. (A) and (B): Summed differences (weeks 9–31) between the simulation results before and after modifying the 2014 and 2015 data, respectively.

**Fig 8:**
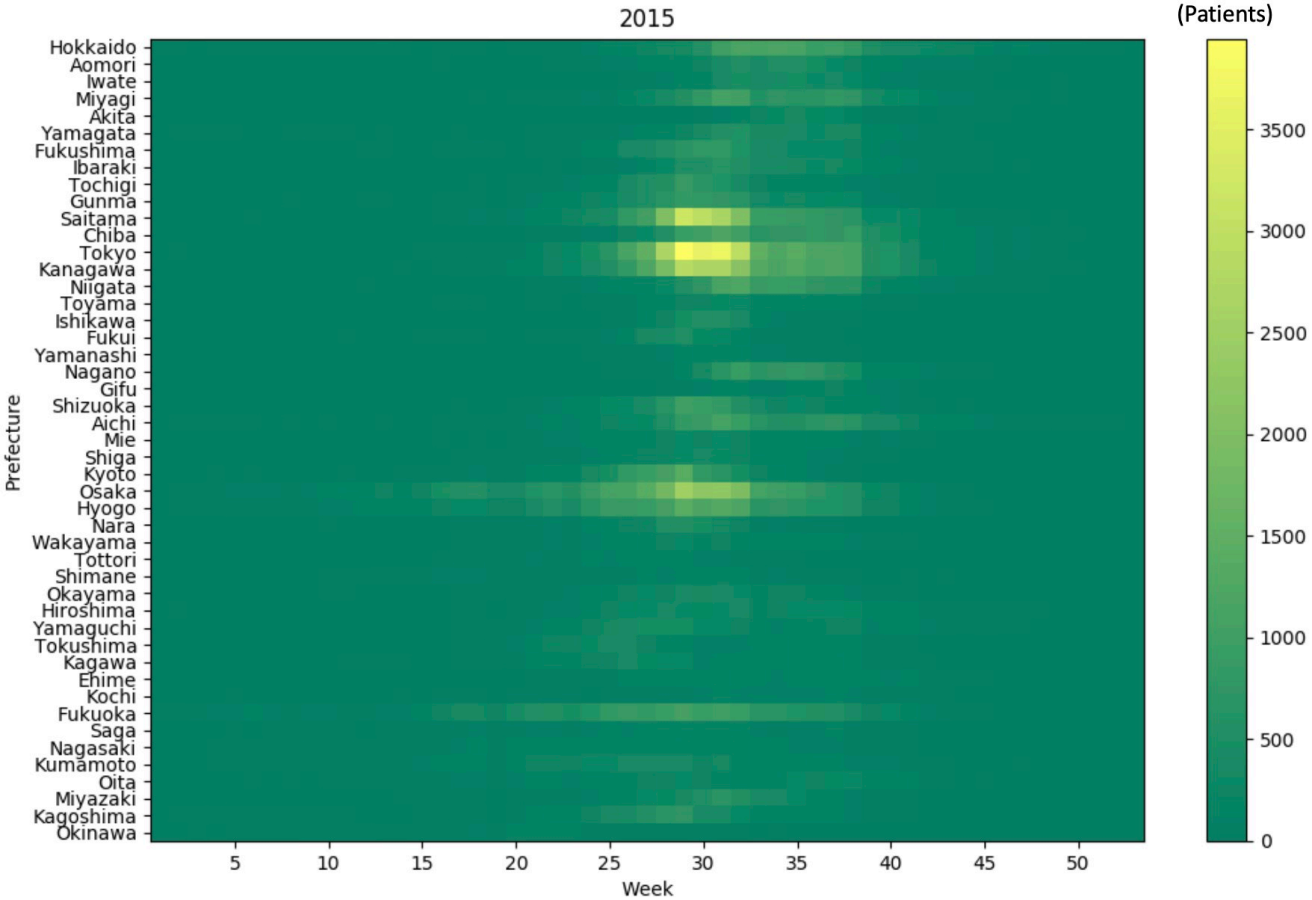
Heat map of the actual HFMD patient numbers in 2015. The actual patient numbers by prefecture were displayed.

To verify the size of the influence of each prefecture’s epidemic against the nationwide outbreak, we investigated how the LSTM model, which contained the machine learning algorithm, learned the number of HFMD patients in each prefecture. Here, we modified the numbers of HFMD patients in each prefecture in 2014 (a small-scale season) and 2015 (a large-scale season) obtained from the IDWR reports before input to the models, and inputted the modified data to the three independently trained models. The total simulated numbers of HFMD patients based on the modified 2014 and 2015 data were compared with the simulation results (weeks 9–31) based on the unmodified data. After summing the differences between the results obtained before and after modifying the input data, we found that among 47 prefectures, replacing the number of patients in Osaka Prefecture maximized the variability of the simulation. The results of other urban areas (Tokyo, Aichi, and Fukuoka) were less variable than those of Osaka (Figs 6 and 7). Moreover, the patient numbers began increasing earlier in Osaka Prefecture than in other prefectures during the actual HFMD outbreak of 2015 (Fig 8). This result indicates that the machine learning algorithm detected the increasing pattern in Osaka as a characteristic feature of large-scale epidemics.

## 4. Discussion

We trained LSTM models to simulate the epidemic patterns of HFMD based on IDWR data in Japan. The models were trained on the HFMD epidemic data of 1999–2013 and simulated the epidemic patterns in 2014–2017 before they were revealed. Furthermore, the models predicted the HFMD epidemic patterns of 2018 and 2019.

HFMD cases in Japan are caused by various types of enteroviruses. The major causative agents were EV-A71 and CV-A16, but the number of cases attributed to CV-A6 has increased since 2011, implying an epidemiological change around 2010 [35]. CV-A6 mainly contributed the large-scale epidemics in 2011, 2013, 2015, and 2017. In a previous study, the dynamics of numbers of patients infected by different type of enteroviruses were explored [36], while the present study aimed to predict an HFMD outbreak, regardless of enterovirus type. Additionally, the learning step of LSTM model included the period when EV-A71, CV-A16, or CV-A6 was major pathogen (i.e., the 1999–2013 season), therefore the learned LSTM model could predict each number of reported HFMD patients, who were infected with enteroviruses including the types isolated until 2013. As the infected patients and epidemic pattern were changed by reflecting the epidemic situation of each type of enteroviruses, the epidemic situations were also reflected into reported HFMD patients and the model. Additionally, it was considered that the LSTM model learned the change of the major pathogen with CV-A6 has increased since 2011 by forget gate in the model. By using the learned LSTM model, the HFMD outbreak in 2019 was showed at Fig 4.

To understand the epidemic patterns of HFMD, we employed LSTM because this algorithm has been widely applied in predictions of influenza, chicken pox, scarlet fever, and malaria [37–40]. It was indicating that use of LSTM model was appropriate for predicting HFMD epidemic pattern.

Recently, LSTM-based simulations of HFMD epidemics have also been reported in China [21–23]. These reports applied an LSTM model with a forget gate and connected cell conditions [21], or with a forget gate [22, 23], the availability of these models was showed. In this study, we used the same model at Gu’s and Zhang’s reports [22, 23], and the LSTM model showed similar performance when the epidemiological data of HFMD in Japan was used. Moreover, HFMD epidemics in Beijing have also been predicted by an algorithm with a gated recurrent unit (GRU) [24], suggesting that the algorithm using GRU was available as same as the LSTM algorithm for predicting the HFMD epidemic. Whereas the yearly patient numbers have remained fairly stable in China, large and small-scale epidemics in Japan have repeated at two-yearly intervals since 2011. Therefore, by predicting the epidemic scales of HFMD using the LSTM model based on IDWR data, we could possibly implement preventive measures at pediatric hospitals, clinics, and communities against large HFMD outbreaks.

The machine learning analysis revealed a unique feature of the Japanese HFMD data, namely, that the HFMD patients in Osaka Prefecture most affected the computational simulation results. With a conventional method, new characteristic in statistical data was found through human, although the LSTM model investigated the new characteristic automatically using “auto-update” learning algorithm. Moreover, we found out the characteristic of Osaka’s data in the process of using the LSTM model. It was indicating that new epidemiological characteristic, which could not be found out through human, could be sought by unpresented approach in epidemiology.

Previous studies showed the prediction of HFMD epidemics at some prefectures in Japan, using the latitude-based approach [27], or the maximum entropy method and the least squares fitting method [28]. These studies compared the results of some prefectures calculated under the same conditions, while in this study, we compared the actual and hypothetical HFMD epidemics, which the number of patients at each prefecture was changed, of the large-scale epidemic. The variability of the simulation was maximum when the number of patients of Osaka prefecture in 2014 and 2015 were replaced.

In this study, the characteristic of Osaka’s data was revealed only by analyzing the weekly recorded numbers of HFMD patients in each prefecture of Japan, and the LSTM algorithm didn’t use the others factor such as Osaka’s geographical location, population, temperature, or humidity. Therefore, the relationship between the characteristic of Osaka’s data and the factors, except the number of HFMD patients, were indefinite. The fact that Osaka is geographically located in the center of Japan and is the third most populous prefecture in Japan may have had an impact.

In Figs 2–4, and S1 Fig, we compared the actual number of patients and the results estimated by LSTM model. By using the data in Figs 2–4, and S1 Fig, RMSE and R^2^ were calculated for evaluating the accuracy of the simulation results. The values of R^2^ from 2014 and 2016’s simulation in Fig 3 were minus because it was suggested that the small-scale epidemic in 2014 and 2016 provided the trend, that the residual sum of squares was larger than the total sum of squares. Additionally, we compared relatively the estimated results at week 25 in Fig 4 for predicting the scale of epidemics in 2018 and 2019.

## 5. Conclusion

In this study, we predicted HFMD outbreaks in Japan using LSTM algorithm. We showed the dynamics of HFMD epidemic 4 weeks later from the numbers of weekly HFMD cases reported IDWR of the National Institute of Infectious Diseases. We predicted HFMD epidemic in 2018 and 2019, and the results of the prediction and the actual reported patients were almost match. Additionally, we found the increasing pattern in Osaka was the characteristic feature of large-scale epidemics.

This study is available for the control of HFMD outbreaks.

## Supporting information

S1 Method Simulation of HFMD patients 2–5 weeks later. (DOCX)

S1 Fig Simulation of HFMD patients 2–5 weeks later in 2015. The LSTM model was trained on the same data as Fig 2. The model was trained on the data of consecutive five weeks and outputted the total number of HFMD patients 2–5 weeks later. The projected numbers of patients 2–5 weeks later were simulated by the same training approach (one week later: interval 0 in Fig 2; two weeks later: interval 1; three weeks later: interval 2; four weeks later: interval 3; five weeks later; interval 4). The input was normalized and standardized. The maximum and minimum output values at interval 0 were adjusted to the maximum and minimum number of patients in 2015, respectively.
(TIF)

S1 Table RMSEs of the simulation results 2–5 weeks later. (DOCX)

S2 Table R^2^ of simulations 2–5 weeks later. (DOCX)
