# Supplementary material for: Prediction of hand, foot, and mouth disease epidemics in Japan using a long short-term memory approach
Source: PLoS One. 2022 Jul 28;17(7):e0271820. doi: 10.1371/journal.pone.0271820 (PMC9333334; doi:10.1371/journal.pone.0271820)
Supplement: S1 Method — (DOCX) [file pone.0271820.s001.docx]

**S1 Method**

**Simulation of HFMD patients 2–5 weeks later**

The LSTM model was trained on the pediatric sentinel data of 1999–2014. After inputting five weeks of data, the trained model applied the method used for generating Fig 1 and outputted the total number of patients 2–5 weeks later. The number of patients 2–5 weeks later in 2015 was simulated by the same learned approach. We checked whether the results of Fig. 1 matched the results of the simulation 2–5 weeks later. The input was normalized and standardized. The maximum and minimum output values of the result in Fig 1 were adjusted to the maximum and minimum numbers of patients in 2015, respectively.
