# Supplementary figures and images for: Prediction of hand, foot, and mouth disease epidemics in Japan using a long short-term memory approach
Source: PLoS One. 2022 Jul 28;17(7):e0271820. doi: 10.1371/journal.pone.0271820 (PMC9333334; doi:10.1371/journal.pone.0271820)

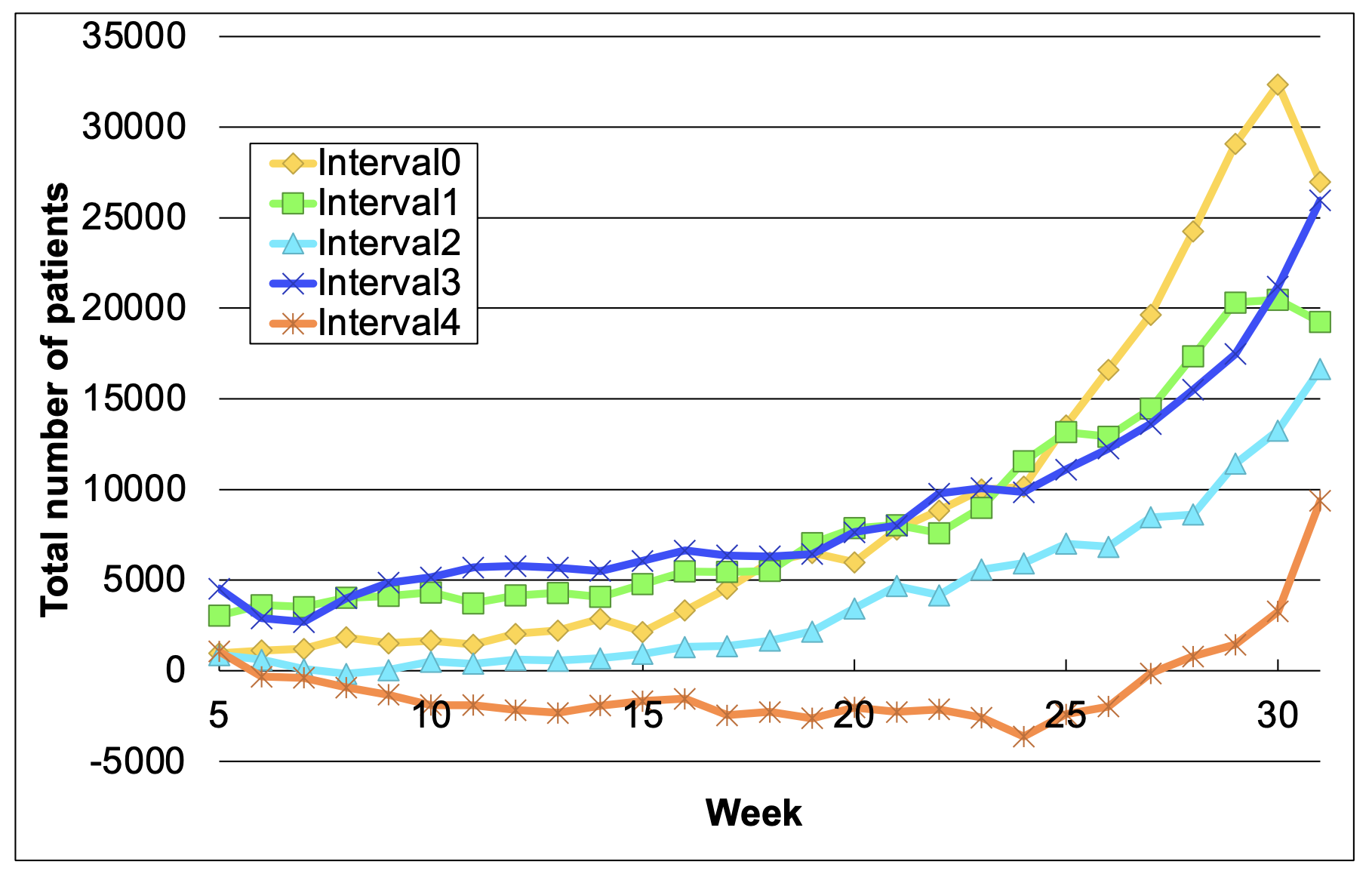

Supplement: S1 Fig — The LSTM model was trained on the same data as Fig 2. The model was trained on the data of consecutive five weeks and outputted the total number of HFMD patients 2–5 weeks later. The projected numbers of patients 2–5 weeks later were simulated by the same training approach (one week later: interval 0 in Fig 2; two weeks later: interval 1; three weeks later: interval 2; four weeks later: interval 3; five weeks later; interval 4). The input was normalized and standardized. The maximum and minimum output values at interval 0 were adjusted to the maximum and minimum number of patients in 2015, respectively. (TIF) [file pone.0271820.s002.tif]
