# Supplementary material for: Prediction of hand, foot, and mouth disease epidemics in Japan using a long short-term memory approach
Source: PLoS One. 2022 Jul 28;17(7):e0271820. doi: 10.1371/journal.pone.0271820 (PMC9333334; doi:10.1371/journal.pone.0271820)
Supplement: S1 Table — (DOCX) [file pone.0271820.s003.docx]

**Table S1. RMSEs of simulations 2–5 weeks later ^a^**

|  | **RMSE** |
| --- | --- |
| **1 week later** | 7.53 × 10^-7^ |
| **2 weeks later** | 1.23 × 10^−6^ |
| **3 weeks later** | 2.06 × 10^−6^ |
| **4 weeks later** | 1.36 × 10^−6^ |
| **5 weeks later** | 4.18 × 10^−6^ |

^a^ Calculated using the summed differences in weeks 5–31.
